# Penetration through Outer Membrane and Efflux Potential in *Pseudomonas aeruginosa* of Bulgecin A as an Adjuvant to β-Lactam Antibiotics

**DOI:** 10.3390/antibiotics12020358

**Published:** 2023-02-09

**Authors:** Choon Kim, Shusuke Tomoshige, Mijoon Lee, Helen I. Zgurskaya, Shahriar Mobashery

**Affiliations:** 1 Department of Chemistry and Biochemistry, University of Notre Dame, Notre Dame, IN 46556, USA; 2 Department of Chemistry and Biochemistry, University of Oklahoma, Norman, OK 73019, USA

**Keywords:** *Pseudomonas aeruginosa*, bulgecin A, β-lactam antibiotics, efflux pumps, porins, potentiation, outer membrane penetration

## Abstract

The treatment of infections by Gram-negative bacteria remains a difficult clinical challenge. In the light of the dearth of discovery of novel antibiotics, one strategy that is being explored is the use of adjuvants to enhance antibacterial activities of existing antibiotics. One such adjuvant is bulgecin A, which allows for the lowering of minimal-inhibitory concentrations for β-lactam antibiotics. We have shown that bulgecin A inhibits three of the pseudomonal lytic transglycosylases in its mode of action, yet high concentrations are needed for potentiation activity. Herein, we document that bulgecin A is not a substrate for pseudomonal efflux pumps, whose functions could have been a culprit in the need for high concentrations. We present evidence that the penetration barrier into the periplasm is at the root of the need for high concentrations of bulgecin A in its potentiation of β-lactam antibiotics.

## 1. Introduction

Bulgecin A, discovered in the 1980s by Takeda Pharmaceutical Company, potentiates the activity of β-lactam antibiotics [1,2]. We documented, by whole genome sequencing of two producer strains, that the cluster of genes for bulgecin biosynthesis is contiguous with that of a β-lactam antibiotic, a likely set up for co-regulation of the production of the two agents concurrently. Furthermore, we have documented that the targets of bulgecin A in *Pseudomonas aeruginosa* are lytic transglycosylases Slt, MltD and MltG [3]. There are 11 lytic transglycosylases in *P. aeruginosa*, the functions of most of which are not fully understood. However, it would appear that inhibition of any of Slt, MltD and MltG is sufficient to predispose bacteria to lysis in the presence of a β-lactam antibiotic, but the effect of bulgecin A on the minimum inhibitory concentration (MIC) values of ceftazidime (CAZ) and meropenem (MEM) has been a modest 2- to 4-fold [3]. Even for this level of activity, high concentrations of bulgecin A are necessary. The question becomes whether bulgecin A is a substrate for bacterial efflux pumps, which would prevent buildup of the compound within the periplasm, or, alternatively, the penetration barrier for bulgecin A is a problem in achieving a sufficient concentration within the periplasm for inhibition of lytic transglycosylases. We report herein that the latter is applicable for bulgecin A. Bulgecin A was synthesized for the investigation reported herein by our reported method [4].

## 2. Results and Discussion

In the present report, we evaluated the effect of bulgecin A on β-lactam MICs with the β-lactam-hypersensitive mutant *P. aeruginosa* K799/Z61, which carries the mutations in *oprM* (efflux), *ampC* (β-lactamase) and *lptE* (lipopolysaccharide transport) genes, and with its parental strain K799/WT [5]. The MICs of CAZ and MEM against both K799/WT and K799/Z61 strains were reduced two-fold by bulgecin A (Table 1). In order to confirm the effect of bulgecin A on the wild-type strain, we examined the β-lactam antibiotics ampicillin (AMP), carbenicillin (CAR) and cefoxitin (FOX), for which *P. aeruginosa* exhibits poor to intermediate susceptibility. The wild-type K799/WT strain showed a 4- to 8-fold decrease in the MIC values of AMP, CAR and FOX in the presence of bulgecin A, while the mutant K799/Z61 exhibited a 2- to 4-fold reduction (Table 1). These results indicate that the OprM is not involved in the transport of bulgecin A.

We measured the MIC of CAZ and MEM against two knockout mutants in the absence and in the presence of bulgecin A (Table 2). The MIC values of MEM were reduced as much as 8- and 4-fold, respectively, for the Δ3-MCS (Δ*mexAB*/Δ*mexXY*/Δ*mexCD*) and Δ6-MCS (Δ*mexAB*-*oprM*/Δ*mexCD*-*oprJ*/Δ*mexEF*-*oprN*/Δ*mexXY*/Δ*triABC*/Δ*mexIJK*) strains (Table 2), compared to the wild-type PAO1. This indicates that MEM may be pumped out through one of three efflux systems (*mexAB*, *mexXY* or *mexCD*). On the other hand, the MIC of CAZ was decreased merely 2-fold against the Δ3-MCS mutant, which implies that CAZ is not a substrate for the efflux pumps deleted in the Δ3-MCS mutant. However, bulgecin A affected only the MIC of MEM, reducing it by 2-fold, regardless of the efflux systems. Therefore, the efflux systems removed in the Δ6-MCS mutant may not be involved in the efflux of bulgecin A.

We further examined 57 transposon-insertion mutants (27 efflux pumps and 29 porins) to identify the pump(s) transporting bulgecin A (Table A1). All transposon mutants were purchased from the Manoil *P. aeruginosa* PAO1 transposon-mutant library [6]. The mutation of the individual gene for the efflux systems and the porin proteins did not significantly affect the susceptibility to CAZ and MEM compared with the wild type in the absence of bulgecin A (Table A1). However, the mutation of *oprM* reduced the MIC of MEM at the highest level (8-fold), indicating that the OprM is a component of an efflux system for MEM. This result is consistent with the reports demonstrating that MEM is a substrate of the MexAB-OprM efflux pump, of which overexpression is responsible for MEM resistance [7,8,9]. Regarding the potentiation activity of bulgecin A for β-lactams, the wild-type strain and most transposon mutants showed merely a two-fold decrease in the MIC values for CAZ and MEM in the presence of bulgecin A (Table A1). The *mexA* and *mexB* mutants exhibited an eight-fold decrease in the MEM MIC in the presence of bulgecin A. On the other hand, bulgecin A raised the MEM MIC by as much as two-fold for the *muxC*, *mexK*, *oprF* and *opdO* mutants. Bulgecin A did not dramatically potentiate the activity of CAZ and MEM against *P. aeruginosa*. We note this in light of the fact that the penetration of small molecules into *P. aeruginosa* is among the most challenging in Gram-negative bacteria; the use of an adjuvant for this pathogen is most warranted [10,11,12,13,14,15].

We also determined the potentiation of bulgecin A (molecular weight 551 Da) for the strains overexpressing FhuA, which is an outer-membrane iron transporter and takes up small molecules up to 600 Da, including some antibiotics in *Escherichia coli* [16,17]. The MICs of all β-lactams against the hyperporinated strains were significantly decreased by as much as 8–32 fold compared with their parental strains, except for MEM (Table 3). Bulgecin A lowered the MIC by another 2- to 4-fold for all β-lactams tested against the FhuA-overexpressing strains. This indicates that bulgecin A does not penetrate the outer membrane of *P. aeruginosa* through FhuA, as the results were comparable to those reported above.

**Figure 1:**
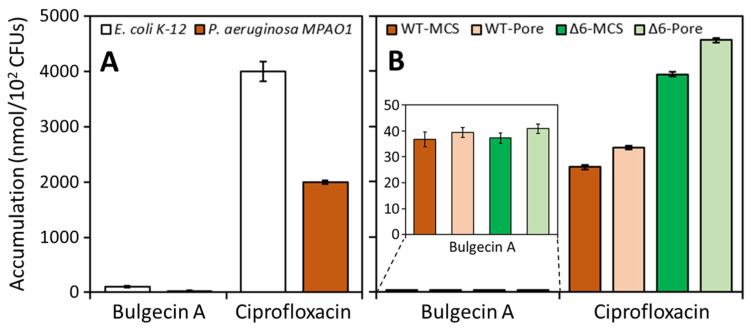
Accumulation of bulgecin A and ciprofloxacin. The accumulation was determined in the wild-type *E. coli* K-12 and *P. aeruginosa* MPAO1 (**A**), and in the wild-type *P. aeruginosa* PAO1 and its mutant *P. aeruginosa* strains (**B**). *P. aeruginosa* MPAO1 is the parental PAO1 strain for transposon insertion used in Manoil Lab at University of Washington. The determinations were performed in triplicate independently.

Next, we explored the degree by which bulgecin A penetrates into the periplasmic space, where the target lytic transglycosylases are sequestered. The accumulation assay was performed by published methods [18,19,20,21]. Bulgecin A showed poor accumulation in *P. aeruginosa*, by as much as 67-fold lower compared with a good-penetrating antibiotic, ciprofloxacin [18,19]. Bulgecin A also penetrated *E. coli* much less efficiently compared with ciprofloxacin (40-fold lower) (Figure 1A, Table A2 for actual numbers). Moreover, we examined the penetration of bulgecin A in the Δ6 and the hyperporinated *P. aeruginosa* strains. The accumulation of ciprofloxacin was increased by approximately two folds in the Δ6 mutants compared with the wild-type strains. The overexpression of FhuA, a porin, slightly raised penetration of ciprofloxacin (Figure 1B). On the other hand, the accumulation of bulgecin A in the mutants was the same as that in the wild type (inset of Figure 1B). This indicates that accumulation of bulgecin A in *P. aeruginosa* was not affected by either the efflux pumps or the FhuA porin.

## 3. Concluding Remarks

The targets of action for both the β-lactam antibiotics and for bulgecin A are located in the Gram-negative bacterial periplasm. As such, both the antibiotic and the adjuvant need to penetrate the bacterial outer membrane and achieve sufficient concentration to inhibit their respective target enzymes. There are two potential impediments to building bulgecin A concentrations in the periplasm: (1) penetration barrier through the outer membrane and (2) bacterial efflux mechanisms that pump out the compound as it penetrates periplasm. In the present report, we have documented that the former applies and the latter decidedly does not. To put it differently, bulgecin A is not a substrate for the *P. aeruginosa* efflux pumps, which is an advantage. However, the penetration of the compound through the outer membrane into the periplasmic space is modest, as we quantified. A structural variant of bulgecin A with improved penetration into Gram-negative bacteria holds promise for effective potentiation of the activity of β-lactam antibiotics in the treatment of difficult infections.

## 4. Materials and Methods

### 4.1. Bacteria, Media, Growth Conditions and Antibiotics

*Pseudomonas aeruginosa* strains Boston 41501 (ATCC 27853), K799/WT (ATCC 12055) and K799/Z61 (ATCC 35151) were purchased from the American Type Culture Collection (ATCC). ATCC 27853 was used as a quality-control strain for the activity of β-lactam antibiotics. The strains K799/WT and K799/Z61 were used to determine whether OprM and LptE are responsible for the transport of bulgecin A. *P. aeruginosa* MPAO1 (the parental PAO1 strain for transposon insertion) and its transposon mutants were obtained from Manoil Lab at University of Washington in order to expand our investigation on the effect of individual components of efflux pumps and porins on the potentiation of bulgecin A for β-lactam antibiotics ceftazidime and meropenem. *P. aeruginosa* Δ3-MCS (Δ*mexAB*/Δ*mexXY*/Δ*mexCD* knockout mutant harboring an empty vector pLAC-MCS), Δ6-MCS (Δ*mexAB*-*oprM*/Δ*mexCD*-*oprJ*/Δ*mexEF*-*oprN*/Δ*mexXY*/Δ*triABC*/Δ*mexIJK* knockout mutant harboring pLAC-MCS), Δ6-Pore (Δ6 harboring pLAC*fhuA*Δ*C*Δ*4L*), PAO-MCS (PAO1 carrying pLAC-MCS), and PAO-Pore (PAO1 harboring pLAC*fhuA*Δ*C*Δ*4L*). The strains Δ3-MCS and Δ6-MCS were utilized to evaluate if any set of efflux pumps would prevent bulgecin A from the accumulation in the periplasm. The strains PAO-Pore and Δ6-Pore were investigated to check whether bulgecin A would enter the cell through a transporter protein FhuA that was overexpressed in the presence of 0.1 mM IPTG [16,17]. Professor Karen Bush at Indiana University kindly gifted us the *P. aeruginosa* Δ*oprD* strain. All strains were grown at 37 °C in LB (Miller; Thermo-Fisher Scientific, Waltham, MA, USA) for overnight cultures and in cation-adjusted Müeller Hinton broth (CAMHB) for antimicrobial susceptibility and potentiation tests. Bulgecin A was purchased from Takeda Pharmaceutical Company, Japan. Ampicillin, carbenicillin, cefoxitin, ceftazidime, ciprofloxacin were purchased from Sigma-Aldrich (St. Louis, MO, USA). Meropenem was bought from the U.S. Pharmacopeia (USP). IPTG, Silicone oil AR20 and High Temperature silicone oil were purchased from Sigma-Aldrich.

### 4.2. Antimicrobial Susceptibility and Potentiation Tests

The minimum inhibitory concentration (MIC) was determined using the broth microdilution method with CAMHB on 96-well plates following CLSI guidelines [22]. The potentiation effect of bulgecin A on β-lactam antibiotics were evaluated by measuring the MIC values of β-lactam drugs in the presence of bulgecin A at the concentration of 100 μg/mL. Bulgecin A was used at 50 μg/mL to screen any efflux pumps or porins that would be involved in the transport of bulgecin A. All experiments were conducted at least in two repeats.

### 4.3. Accumulation of Bulgecin A

**Figure A1:**
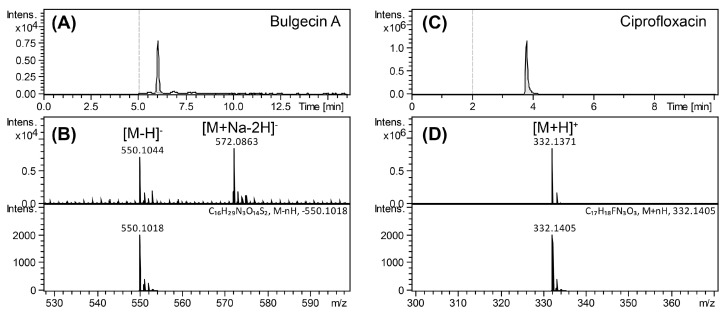
Representative LC/MS traces of bulgecin A and ciprofloxacin. The extracted-ion-chromatograms (EIC)s (**A**,**C**) and corresponding mass spectra (**B**,**D**) are given. For mass spectra, top panels show mass spectra of samples and bottoms are theoretical ones.

The accumulation of bulgecin A was measured by following the protocol previously published with slight modification [21,23]. Briefly, the cell pellets were resuspensed in pre-warmed 1 × PBS to ~10^10^ CFU/mL. One milliliter of the cell suspension was dispensed into each well of a 24-well plate. Bulgecin A and ciprofloxacin were added into each well to be the final concentration of 50 μM. Ciprofloxacin, which well penetrates *P. aeruginosa*, was used as a positive control [18,19]. The colony forming units (CFUs) of each sample were measured immediately after incubation at 37 °C for 15 min with gentle shaking. Each sample (800 μL) was carefully layered on 600 µL of silicone oil (3:7 mix of AR20:High Temperature; density of 1.03; cooled to −80 °C) in an 1.7-mL microcentrifuge tube. The compounds that were not penetrated were separated by pelleting the bacteria through silicone oil at 13,000× *g* for 5 min at room temperature. After removing the supernatant and the silicone oil, bulgecin A and ciprofloxacin were extracted from *P. aeruginosa* by three cycles of Freeze-Thaw with a dry ice/ethanol bath and a 65 °C water bath. The extracted compounds were centrifuged at 20,000× *g* for 15 min at room temperature to remove the residual bacterial debris, followed by drying the supernatants with Genevac miVac Centrifugal Concentrator (Genevac, Ipswich, UK) at room temperature for 10 h. Finally, bulgecin A and ciprofloxacin were resuspended in 200 μL of water:acetonitrile (50:50) and water: acetonitrile (95:5), respectively, followed by LC/MS analyses. Quantification of ciprofloxacin using LC/MS was described previously [23]. For bulgecin A, the following LC gradient was used on a Waters Acquity UPLC BEH Amide column (2.1 × 150 mm, 1.7 μm): 2 min at 20 A:80 B, 11 min to 80 A:20 B, 0.1 min to 20 A:80 B; 2 min at 20 A:80 B where A = 0.1% formic acid in water, B = 0.1% formic acid in acetonitrile. Representative LC/MS traces of ciprofloxacin and bulgecin A are given in Figure A1.

## Figures and Tables

**Table 1:** Effect of bulgecin A on the MIC of β-lactams against a hypersensitive *P. aeruginosa* strain.

	MIC (μg/mL) (Fold Change) ^a^										
Antibiotic ^b^	Bulgecin A	CAZ		MEM		AMP		CAR		FOX	
Bulgecin A ^c^		−	+	−	+	−	+	−	+	−	+
ATCC 27853 ^d^	>256	2	0.5 (4)	0.25	0.125 (2)	2048	512 (4)	64	32 (2)	2048	1024 (2)
K799/WT	>256	1	0.5 (2)	0.5	0.25 (2)	2048	256 (8)	128	32 (4)	2048	512 (4)
K799/Z61 ^c^	>256	0.5	0.25 (2)	0.5	0.25 (2)	0.25	0.06 (4)	0.125	0.03 (4)	0.5	0.25 (2)

^a^ The fold change of the MIC in the presence of bulgecin A is indicated in the brackets. ^b^ AMP, ampicillin; CAR, carbenicillin; CAZ, ceftazidime; FOX, cefoxitin; MEM, meropenem. ^c^ The concentration of bulgecin A was 100 µg/mL. ^d^ A reference strain ATCC27853 was used as a quality control of the antibacterial susceptibility. The experiments were performed in triplicate independently.

**Table 2:** Effect of bulgecin A on the MIC of CAZ and MEM against efflux pump- or porin-knockout mutants.

	MIC (µg/mL) (Fold Change)			
β-Lactam	CAZ		MEM	
Bulgecin A ^a^	−	+	−	+
PAO1	2	1 (2)	1	0.5 (2)
Δ3-MCS ^b^	0.5	0.5 (1)	0.125	0.06 (2)
Δ6-MCS ^b^	1	0.5 (2)	0.25	0.125 (2)

^a^ Bulgecin A was used at 100 μg/mL. ^b^ Δ3-MCS, Δ*mexAB*/Δ*mexXY*/Δ*mexCD* knockout mutant harboring an empty plasmid pLAC-MCS; Δ6-MCS, Δ*mexAB*-*oprM*/Δ*mexCD*-*oprJ*/Δ*mexEF*-*oprN*/Δ*mexXY*/Δ*triABC*/Δ*mexIJK* knockout mutant carrying pLAC-MCS. The experiments were performed in triplicate independently.

**Table 3:** Effect of bulgecin A on the MIC of β-lactams against the strains overexpressing a channel protein FhuA ^a^.

	MIC (μg/mL) (Fold Change)									
β-Lactam	CAZ		MEM		AMP		CAR		FOX	
Bulgecin A ^b^	−	+	−	+	−	+	−	+	−	+
*P. aeruginosa* PAO1										
PAO-MCS ^c^	2	1 (2)	0.5	0.25 (2)	2048	1024 (2)	64	32 (2)	2048	1024 (2)
PAO-Pore ^c^	0.06	0.03 (2)	0.25	0.06 (4)	128	64 (2)	8	4 (2)	256	64 (4)
Δ6-MCS ^c^	1	0.5 (2)	0.25	0.125 (2)	512	256 (2)	2	1 (2)	1024	512 (2)
Δ6-Pore ^c^	0.06	0.03 (2)	0.125	0.03 (4)	16	4 (4)	0.25	0.125 (2)	64	32 (2)

^a^ The MIC was determined in the presence of 0.1 mM IPTG to induce the overexpression of FhuA. ^b^ The concentration of bulgecin A was 100 μg/mL. ^c^ PAO-MCS, *P. aeruginosa* PAO1 harboring pLAC-MCS; PAO-Pore, PAO-MCS harboring pLAC*fhuA*Δ*C*Δ*4L* plasmid overexpressing FhuA; Δ6-MCS, Δ*mexAB*-*oprM*/Δ*mexCD*-*oprJ*/Δ*mexEF*-*oprN*/Δ*mexXY*/Δ*triABC*/Δ*mexIJK* knockout mutant harboring pLAC-MCS; Δ6-Pore, Δ6-MCS harboring pLAC*fhuA*Δ*C*Δ*4L*. The experiments were conducted three times independently.

## Data Availability

Not applicable.

## Appendix A

**Table A1:** Effect of bulgecin A on the MICs of CAZ and MEM against efflux pump- or porin-transposon mutants.

Strain	MIC (µg/mL)				Strain	MIC (µg/mL)			
	CAZ		MEM			CAZ		MEM	
Bulgecin A ^a^	−	+	−	+	Bulgecin A ^a^	−	+	−	+
MPAO1 ^b^	2	1	2	1	PW1427 (*opdF*)	2	1	1	1
PW1778 (*mexA*)	1	1	0.5	0.125	PW1873 (*oprM*)	0.5	0.5	0.25	0.25
PW1781 (*mexB*)	1	0.5	0.5	0.125	PW2374 (*opdH*)	2	1	1	1
PW1265 (*triA*)	2	1	1	0.5	PW2855 (*opdD*)	1	1	1	0.5
PW1267 (*triB*)	2	1	1	0.5	PW3127 (*oprH*)	2	1	1	1
PW1271 (*triC*)	2	1	1	0.5	PW4134 (*oprF*)	2	1	2	2
PW3229 (*PA1237*)	2	1	1	0.5	PW4636 (*opdO*)	2	1	1	2
PW3231 (*PA1238*)	2	1	1	0.5	PW4767 (*opdG*)	2	1	1	1
PW3608 (*mexM*)	2	1	1	0.5	PW5076 (*opdJ*)	2	1	1	0.5
PW3611 (*mexN*)	2	1	1	0.5	PW5187 (*oprN*)	2	1	2	1
PW5222 (*czcA*)	2	1	4	2	PW5196 (*opdT*)	2	1	1	0.5
PW5224 (*czcB*)	2	1	1	0.5	PW5232 (*opmB*)	2	1	1	0.5
PW5226 (*czcC*)	2	1	1	0.5	PW5526 (*opdB*)	2	1	1	0.5
PW5233 (*muxC*)	2	1	2	2	PW5621 (*oprQ*)	2	1	1	0.5
PW5235 (*muxB*)	2	1	1	0.5	PW6095 (*opdQ*)	2	1	1	0.5
PW5237 (*muxA*)	2	1	1	0.5	PW6333 (*oprB*)	2	1	1	0.5
PW6963 (*mexQ*)	2	1	1	0.5	PW6504 (*oprP*)	2	1	1	0.5
PW6965 (*mexP*)	2	1	1	0.5	PW6506 (*oprO*)	2	1	1	0.5
PW7218 (*mexK*)	2	1	2	2	PW7089 (*opdR*)	2	1	1	0.5
PW7220 (*mexJ*)	2	1	1	1	PW7416 (*oprC*)	2	1	1	0.5
PW8135 (*mexH*)	2	1	1	0.5	PW7874 (*oprG*)	2	1	1	0.5
PW8137 (*mexI*)	2	1	1	0.5	PW8010 (*opdL*)	2	1	1	0.5
PW8385 (*mexV*)	1	1	1	0.5	PW8084 (*opdN*)	2	1	1	0.5
PW8390 (*mexW*)	1	1	1	0.5	PW8139 (*opmD*)	2	1	2	0.5
PW8750 (*mexD*)	2	1	1	0.5	PW8575 (*opdP*)	2	1	1	0.5
PW8751 (*mexC*)	2	1	1	1	PW8748 (*oprJ*)	2	1	1	0.5
PW9677 (*PA5159*)	2	1	1	0.5	PW9244 (*opdK*)	2	1	1	0.5
PW9679 (*PA5160*)	1	1	1	0.5	PW9369 (*opmH*)	2	1	2	1
PW1276 (*opdC*)	2	1	2	1	Δ*oprD* ^c^	2	1	2	2

^a^ Bulgecin A was used at 50 μg/mL. ^b^ MPAO1 is the parental *P. aeruginosa* PAO1 for the transposon mutants generated by the Manoil Lab at University of Washington. ^c^ Δ*oprD* is the *oprD*-knockout mutant. The determination was performed in duplicate.

**Table A2:** Accumulation of bulgecin A in the wild-type *E. coli* K-12 and *P. aeruginosa* MPAO1, and the mutant *P. aeruginosa* strains.

Strain	Accumulation (nmol/10^12^ CFUs) ^a^	
	Bulgecin A	Ciprofloxacin ^b^
*E. coli* K-12	100 ± 17	4000 ± 180
*P. aeruginosa* MPAO1	30 ± 3	2000 ± 32
*P. aeruginosa* PAO1		
PAO-MCS	37 ± 3	2300 ± 44
PAO-Pore	39 ± 2	2600 ± 35
Δ6-MCS	37 ± 2	4000 ± 33
Δ6-Pore	41 ± 2	4600 ± 38

^a^ The amounts of the compounds were normalized by dividing with the same CFU. ^b^ Ciprofloxacin was used as a reference antibiotic for the accumulation assay. The experiments were performed in triplicate independently.
